# Künstliche Intelligenz bei der Diagnose Seltener Erkrankungen: die Entwicklung der Phänotyp-Analyse

**DOI:** 10.1007/s00103-022-03602-2

**Published:** 2022-10-24

**Authors:** Peter M. Krawitz

**Affiliations:** Institut für Genomische Statistik und Bioinformatik, Universitätsklinikum Bonn, Rheinische-Friedrich-Wilhelms-Universität Bonn, Venusberg Campus 1, 53127 Bonn, Deutschland

**Keywords:** Künstliche Intelligenz, Maschinelles Lernen, Seltene Erkrankungen, Syndromologie, Faziale Gestalt, Artificial intelligence, Machine learning, Rare disorders, Dysmorphology, Facial gestalt

## Abstract

Durch die Analyse des Erscheinungsbildes (Phänotyp) von Patient:innen kann die Diagnostik von Seltenen Erkrankungen unterstützt werden, da bei vielen genetischen Erkrankungen charakteristische Abweichungen (Dysmorphologien) auftreten. Diese betreffen z. B. die Merkmale des Gesichts – die „faziale Gestalt“.

In diesem Beitrag wird ein Bereich der künstlichen Intelligenz (KI) beleuchtet, in dem es in den letzten Jahren große Fortschritte gegeben hat: die Erkennung charakteristischer Muster in medizinischen Bilddaten mittels vielschichtiger, gefalteter künstlicher neuronaler Netzwerke (Next-Generation Phenotyping – NGP). Die technischen Grundlagen der Methode werden kurz beschrieben und es wird auf die hohe Relevanz von frei zugänglichen Daten für die Wissenschaftsgemeinschaft zur Entwicklung von KI eingegangen. Des Weiteren wird erläutert, warum Entscheidungen von KI immer nachvollziehbar bleiben sollten und wie es gelingen kann, die Herausforderungen in Hinblick auf Datenschutz und Transparenz zu meistern.

Zukünftig können Software-Anwendungen mit KI ärztliche Fachkräfte bei der Diagnostik von Seltenen Erkrankungen unterstützen. Das Vertrauen in den Einsatz von KI wird steigen, wenn Patient:innen ihre Datenhoheit behalten und nachvollziehen können, auf welchem Weg die Diagnose entstanden ist.

## Abstract (en)

Rare diseases can often be diagnosed by carefully assessing the phenotype of the patient, as characteristic deviations (dysmorphisms) occur in many genetic diseases. These affect, for example, the features of the face – the “facial gestalt.”

This paper highlights an area of artificial intelligence (AI) in which there has been great progress in recent years: the recognition of characteristic patterns in medical image data using deep, convolutional neural networks (next-generation phenotyping – NGP). The technical basis of the method is briefly described and the high relevance of FAIR data for the scientific community to develop AI is discussed. Furthermore, it is explained why decisions made by AI should always remain comprehensible and how it can overcome the challenges with regard to data protection and transparency.

In the future, software applications with AI will support medical professionals in the diagnosis of rare diseases. AI will be trustworthy if patients retain their data sovereignty and can understand how the diagnosis was made.

## Einleitung

Der Begriff „Blickdiagnose“ besagt, dass durch alleinige Inspektion des Erscheinungsbildes (Phänotyp) auf eine zugrunde liegende Erkrankung geschlossen werden kann. Bei Syndromen, also einer bestimmten Konstellation von Anomalien, reicht oftmals schon eine Abweichung in der Struktur und Form (Dysmorphologie) des Gesichts für eine Blickdiagnose. Aufgrund seines hohen Informationsgehalts wird das Gesicht (die Fazies) manchmal auch als für eine bestimmte Erkrankung charakteristische „faziale Gestalt“ bezeichnet – ein Fachbegriff, der auch in der englischsprachigen Literatur verwendet wird („facial gestalt“). Interessanterweise wird dieser Befund jedoch manchmal gar nicht näher beschrieben. Die Fachliteratur der letzten Jahre, in der über viele Gen-Phänotyp-Assoziationen berichtet wurde, ist inzwischen aber voll von „novel syndromes with intellectual disability with a characteristic facial gestalt“. Eine Suche mit dem Terminus „abnormal facial shape“ in der Human Phenotype Ontology (HPO), die dieses Wissen strukturiert, liefert bereits Verknüpfungen mit über tausend Genen^1^ [1]. Ein Beispiel für eine Seltene Erkrankung mit markanten Gesichtsmerkmalen ist das Kabuki-Syndrom, benannt nach einer traditionellen japanischen Theaterform mit spezieller Schminktechnik. Bei Abweichungen von der Norm sollte aber immer an eine Glockenkurve gedacht werden. Vieles kann abweichend sein, aber nur manches ist so klar, dass es namensgebend ist.

Hieraus kann man zweierlei ableiten: 1) Es ist möglich, präzise mit phänotypischer Evidenz zu diagnostizieren, aber 2) die sprachliche Begründung einer Entscheidung ist schwer. Der Hinweis auf die informative Region im Gesicht kann zwar richtig, aber für eine dysmorphologisch unkundige Person dennoch nicht informativ sein. In diesem Beitrag soll u. a. gezeigt werden, dass dies ebenso für computergestützte Verfahren in der Dysmorphologie gilt. Ein Teilbereich der Informatik, der sich mit der Automatisierung intelligenten Verhaltens bzw. komplexer Entscheidungsstrukturen beschäftigt, versucht den Prozess der Blickdiagnose abzubilden. In Anlehnung an den positiv besetzten Begriff des *Next-Generation Sequencing *(NGS), der in den letzten Jahren genomweite Analysen ermöglichte, wird der Einsatz von künstlicher Intelligenz (KI) bei der Phänotyp-Analyse in der englischsprachigen Literatur häufig auch mit *Next-Generation Phenotyping *(NGP) bezeichnet. Wie die Erfolge der letzten Jahre zeigen, lässt sich durch den Einsatz von NGP ebenfalls die Effizienz in der Diagnostik steigern. Dies ist insbesondere für den Bereich der Seltenen Erkrankungen von Bedeutung, da hier viele Ärzt:innen Diagnosen nur einmalig in ihrem Berufsleben stellen können, obwohl das „Muster“ erkennbar ist.

In diesem Beitrag werden Methoden und Ergebnisse medizinischer KI erläutert. Es wird zunächst die Funktionsweise künstlicher neuronaler Netzwerke beschrieben, die sich am biologischen Vorbild orientiert. Um die Leistungsfähigkeit von KI einordnen zu können, wird erklärt, wie man diese bestimmt. Danach wird auf die Herausforderung geeigneter Datensammlungen eingegangen. Zuletzt wird ein Ausblick auf mögliche Entwicklungen in der Zukunft gegeben.

## Technische Grundlagen

Wenn in den letzten Jahren von KI die Rede ist, wird damit auf technischer Ebene meist die Mustererkennung mittels vielschichtiger künstlicher neuronaler Netzwerke mit Faltungscodierung (*Convolutional Neural Networks – CNN*) gemeint. Die Architektur ähnelt dabei rezeptiven Feldern im visuellen Kortex des Gehirns. Unter „Faltung“ versteht man eine mathematische Operation, bei der zum Beispiel aus 9 Pixelwerten eines Quadrats in einer Bilddatei ein Mittelwert gebildet wird. Auf diese Weise kann ein Neuron die Information der lokalen Umgebung verarbeiten.

Das Training dieser Netzwerke mittels annotierter Bilddaten wird auch als *Deep Learning* („tiefes Lernen“) bezeichnet. Am Ende des Trainingsprozesses wird der Erfolg anhand eines unabhängigen Testdatensatzes überprüft. Es kann zum Beispiel gemessen werden, wie häufig die richtige Diagnose unter 10 vorgeschlagenen Differentialdiagnosen vorkommt (Top-10-accuracy Rate). Bei der Beurteilung der Güte eines Klassifikators sollte die erzielte Genauigkeit in Bezug gesetzt werden zu dem Wert, der zufällig erwartet würde. Wenn also ein System eine Unterstützung in der Differentialdiagnostik für 1000 Syndrome bietet und eine Top-10-accuracy von 50 % erreicht, dann wäre dies circa 50-mal besser, als zufällig bei gleich verteilten Daten zu erwarten wäre. Tatsächlich sind dies ungefähr die Werte, die die KI „GestaltMatcher“ erzielt [2]. GestaltMatcher ist ein Open-Source-Software-Projekt und gehört aktuell zu den leistungsfähigsten im Bereich NGP.

Für die Durchbrüche im Bereich des tiefen Lernens in den letzten Jahren gibt es im Wesentlichen 2 Gründe. Zum einen profitieren die rechenintensiven Trainingsprozesse der künstlichen neuronalen Netzwerke erheblich von modernen Grafikkarten und zum anderen stehen für viele medizinische Fragestellungen mittlerweile große Bilddatensammlungen für das Training zur Verfügung. *Deep Learning* und *Big Data *sind eng miteinander verknüpft. Als einfache Daumenregel gilt: Man benötigt mindestens 10-mal so viele Datensätze wie zu trainierende Parameter [3]. In einem vielschichtigen Netzwerk mit Hunderten Knoten und Tausenden zu gewichtenden Verbindungen entspricht dies üblicherweise einer Zahl im fünf- bis sechsstelligen Bereich.

Gerade bei Seltenen Erkrankungen ist es jedoch oft nur möglich, Bilddaten von Patient:innen im zwei- bis dreistelligen Bereich zu sammeln. Damit tiefes Lernen dennoch funktioniert, muss ein Trick angewendet werden, der auch als *Transfer Learning* („Übertragungslernen“) bezeichnet wird. Dabei wird nicht mit einem zufällig initialisierten Netzwerk, also bei „null“ angefangen, sondern die Gewichte werden aus einem Netzwerk übernommen, das ein ähnliches Problem lösen kann. Die Analyse von Porträtbildern syndromaler Patient:innen baut auf Netzwerken auf, die auch zur Erkennung nicht betroffener Personen verwendet werden können (Gesichtserkennungssoftware). Diese Netzwerke werden anhand von Millionen Porträtbildern trainiert und können unter anderem für die Entsperrung von Smartphones eingesetzt werden. Für die dysmorphologische Fragestellung wird ein solches Netzwerk um eine weitere Schicht ergänzt und es werden dieser letzten Schicht, den „Neuronen“ oder „Knoten“, die nun zu erlernenden Erkrankungen zugeordnet. Durch diesen Wissenstransfer ist das Netzwerk bereits für wichtige Strukturen im Gesicht, wie Nase, Mund und Augenbrauen, sensibilisiert und kann schneller und mit weniger Anschauungsbeispielen charakteristische Abweichungen von der Norm lernen.

*Transfer Learning* kann auch auf weitere Körperstrukturen angewendet werden, wenn es z. B. darum geht, mit wenig Datensätzen von Personen mit Seltenen Erkrankungen zu arbeiten. Wenn eine KI beispielsweise für angeborene Retinopathien trainiert werden soll und nur einige Hundert Datensätze von Funduskopien (Augenspiegelungen) zur Verfügung stehen, kann auf Netzwerken aufgebaut werden, die für die Einstufung der altersbedingten Makuladegeneration entwickelt wurden. Hierfür gibt es in der UKBiobank des Vereinigten Königreichs viele Tausend Datenpunkte und die Bedeutung dieser Biobanken wird für die Entwicklung medizinischer KI weiter steigen.

## Der klinische Merkmalsraum

**Figure 1:**
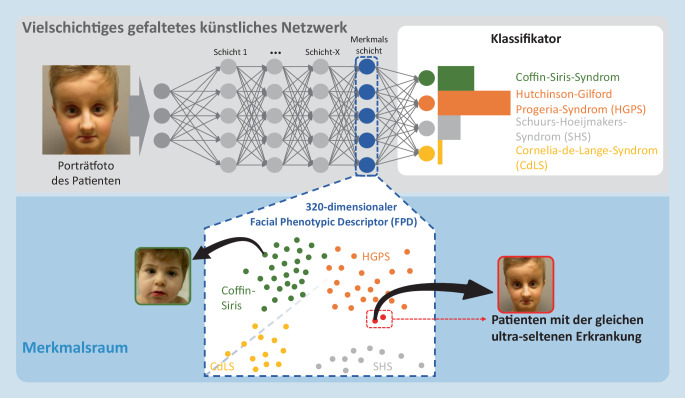

Ein Netzwerk, das gelernt hat eine große Anzahl unterschiedlicher Syndrome zu erkennen, kann auch als mathematische Abbildungsfunktion verstanden werden (Abb. 1). Einem Bild, das dem Netzwerk präsentiert wird, kann ein Ort oder eine Koordinate in einem Raum zugeordnet werden, in dem sich wiederum Abstände messen lassen. Da dieser Raum besonders gut zur Klassifizierung von fazialen Dysmorphien geeignet ist, entspricht Nähe in diesem Raum der „syndromalen Ähnlichkeit“. Die vorletzte Schicht im Netzwerk, die auch „Merkmalsschicht“ (*Feature Layer*) genannt werden kann, wird verwendet, um einen Merkmalsraum aufzuspannen. Je näher die Werte eines Knotens für 2 unterschiedliche Bilder beieinanderliegen, desto größer ist die Übereinstimmung für das darüber repräsentierte Merkmal.

Dieses Vorgehen ist mit einer Unterhaltung zwischen Genetiker:innen auf einer Konferenz während der Kaffeepause vergleichbar. Beide haben Patient:innen mit einer unbekannten Erkrankung und sie möchten abgleichen, ob sich die Fälle ähneln. Die Unterhaltung fängt vielleicht damit an, dass sie Differentialdiagnosen nennen, an die sie zunächst gedacht haben, die sich dann aber nicht bestätigen ließen, sodass sie an eine neue Entität denken. Dann klopfen sie Merkmale ab, die insbesondere von den bekannten Erkrankungen abweichen. Vielleicht kommen sie zu dem Schluss, dass gerade die Form der Augenbrauen in der Kombination mit allen anderen Merkmalen, die durchaus Übereinstimmung mit anderen Syndromen liefern können, zur Abgrenzung geeignet sind. In dieser Dimension des Merkmalsraums wären die beiden Fälle dann nah beieinander, aber weit entfernt von bereits bekannten Erkrankungen.

Eine Clusteranalyse kann dann Aufschluss darüber geben, ob es sich um eine Gestalt handelt, die sich signifikant von anderen unterscheidet und damit auch eine noch unbeschriebene molekulare Ursache wahrscheinlich macht. Der Algorithmus GestaltMatcher unterstützt neben der Klassifikation, also der Generierung bekannter Differentialdiagnosen, auch die Clusterbildung und kann deshalb auch zur Analyse von Syndromen genutzt werden, die noch nicht mit einem Gen assoziiert sind [2]. Der GestaltMatcher-Ansatz stellt auf der Ebene des Phänotyps das Pendant zu GeneMatcher dar, der eine Zuordnung von Forschungsfällen auf Genotyp-Ebene ermöglicht.

Das Prinzip des Merkmalsraums, der für klinisch relevante Ähnlichkeitsvergleiche geeignet ist, lässt sich auch auf andere Bildentitäten wie Funduskopien oder Röntgenbilder anwenden. Auch hier können seltene Muster in einem Merkmalsraum geclustert und damit erkannt werden. Speziell angeordnete Flecken auf der Netzhaut (*Fundus flavimaculatus*) erlauben zum Beispiel erfahrenen Augenärzt:innen die Blickdiagnose eines durch Mutationen im ABCA4-Gen verursachten Morbus Stargardt. Und Kinderradiolog:innen können zapfenförmige Epiphysen oder Metaphysen mit flügelförmigen seitlichen Auswüchsen (*Angels*) bei der Beurteilung von Röntgenaufnahmen der Hand wichtige Hinweise auf die genetische Ursache geben [4].

## Weiterentwicklung von KI: frei verfügbare Daten und Erklärbarkeit

Auch die öffentlichen Fördermittelgeber haben erkannt, wie wichtig es ist, die Daten, die zur Entwicklung einer KI verwendet wurden, der Wissenschaftsgemeinschaft zugänglich zu machen. Bilddaten und darauf basierende Ergebnisse sollten ***F****indable*, ***A****ccessible*, ***I****nteroperable* ***R****eproducible* und damit FAIR sein [5]. Für das „überwachte Lernen“ (*Supervised Learning*), bei dem die Ergebnisse des Lernprozesses der KI mit bereits bekannten, richtigen Ergebnissen verglichen werden, können neben der Diagnose auch einzelne, klinische Merkmale verwendet werden. Die sorgfältige Annotation des medizinischen Fachwissens in maschineninterpretierbarer Form ist eine Mammutaufgabe und einer der größten derzeitigen Flaschenhälse bei der Verbesserung von KI.

Faire Daten ermöglichen nicht nur die Reproduzierbarkeit der KI-Ergebnisse, sondern sie ermöglichen auch den Vergleich unterschiedlicher Algorithmen und können bei der Interpretation der Ergebnisse helfen.

Ein eigener Forschungsbereich ist die *Explainable AI* (XAI, „erklärbare künstliche Intelligenz“), in dem versucht wird die Muster in einem Bild sichtbar zu machen, die für die Entscheidung der KI relevant sind. Auch Dysmorpholog:innen können treffsichere Diagnostiker:innen sein, gute Lehrer:innen sind sie erst, wenn sie ihre Entscheidungen auch erklären können. Im Idealfall können die Methoden zur Veranschaulichung des Informationsgehalts auch Fachleuten bei der Reflexion einer Blickdiagnose helfen und damit zu einer präziseren phänotypischen Beschreibung führen.

Angenommen, zusammengewachsene Augenbrauen (Synophrys) sind das charakteristische Merkmal eines Syndroms, dann würde die Vorhersagegenauigkeit dieser Erkrankung durch die KI sinken, wenn im Porträt diese Region überdeckt ist. Indem man den Sensitivitätsabfall bei Verdeckung *(Occlusion Sensitivity Mapping) misst, *kann man genau diese Bereiche ausfindig machen. Man überdeckt hierbei sukzessive alle Teile des Bildes zum Beispiel mit einem kleinen schwarzen Quadrat und misst, wie stark die Performanz abfällt. Es gibt noch weitere, ausgefeiltere Methoden, die mit Gradienten im Netzwerk arbeiten, um die informativen Bildelemente zu veranschaulichen [8].

XAI kann auch dabei helfen Klassifikationsfehler besser zu verstehen. Sie setzt aber üblicherweise voraus, dass die Trainingsdaten einsehbar sind. Ein berühmt-berüchtigtes Beispiel ist eine KI von Google, die zur automatischen Bilderkennung eingesetzt wurde und schwarze Menschen als Gorillas markierte. Die Erklärung hierfür war schnell gefunden, da die KI mit Bilddaten von ausschließlich weißen Menschen trainiert wurde. Wenn die Auswahl der Trainingsdaten nicht repräsentativ ist, also die Wirklichkeit nicht in ihrer Diversität abbildet, können daraus falsche Zuordnungen resultieren [6]. Interessanterweise ist aber die Variabilität des menschlichen Gesichts, die auf unterschiedlichen ethnischen Hintergrund zurückzuführen ist, deutlich geringer als die Variabilität, die durch syndromale Erkrankungen verursacht wird. Es ist daher relativ einfach möglich, vergleichbare Performanzen zu erzielen, indem Trainingsdaten zumindest mit einigen Patient:innen asiatischen oder afrikanischen Ursprungs angereichert werden.

## Der Weg zum Medizinprodukt

Für die Zulassung von KI-Software als Medizinprodukt ist die Erklärbarkeit der Ergebnisse eine wichtige Anforderung [7]. Wenn ein Arzt oder eine Ärztin eine Entscheidung treffen soll, die unter anderem auf den Resultaten einer KI basiert, so muss die KI zumindest versuchen, ihren Befund zu begründen. Wenn der Arzt oder die Ärztin einen Störfaktor (*Confounder*) erkennt, der die KI beeinflusst haben könnte, dann kann die Differentialdiagnose der KI verworfen werden. Lacht der Patient auf der Aufnahme, weil er am Angelman-Syndrom erkrankt ist oder weil sich dies aus der Situation beim Fotografieren ergab? Die Ärztin wird sich bei der Interpretation der Bilder erinnern. Die KI kennt den Kontext nicht. Menschen sind meist besser darin, systematische Verzerrungen (*Bias*) aufzuspüren. Maschinen sind hingegen nicht anfällig für Rauschen (*Noise*). Die Kombination von Mensch und Maschine kann also *Bias* und *Noise* effektiv reduzieren [9].

Mit der Zulassung einer KI als Medizinprodukt geht auch die wirtschaftliche Verwertung dieser Technologie einher. Auch hierbei sollte die Bedeutung FAIRer Daten berücksichtigt werden. Wenn Algorithmen und Daten bei einem Anbieter gebündelt sind, besteht die Gefahr einer Monopolbildung, die letztlich eine technologische Weiterentwicklung behindern wird. Um dem vorzubeugen, wurde die Datensammlung von GestaltMatcher zum Betrieb an die gemeinnützige Arbeitsgemeinschaft für Gen-Diagnostik e. V. übergeben. Dadurch können die Bilddaten mit der wissenschaftlichen Gemeinschaft geteilt und von ihr mittels Human Phenotype Ontology annotiert werden, wenn die Patient:innen dieser Verwendung zustimmen.

Wie bei allen Daten, die das Potenzial der Reidentifizierbarkeit bergen, ist eine effektive Pseudonymisierung nicht möglich und Vertrauen in die KI kann nur durch Transparenz geschaffen werden. Dies ist der Kerngedanke der „dynamischen Einwilligung“ (*Dynamic Consent*), bei der das Studienteam unter Zuhilfenahme digitaler Technologien über die Ziele der Forschung informiert und Teilnehmende einfacher mit den Wissenschaftler:innen interagieren können [10]. Zudem ist in der Europäischen Datenschutzgrundverordnung (EU-GDPR) geregelt, dass jederzeit nachvollziehbar sein muss, wer mit welchen Daten arbeitet, und dass Patient:innen ohne Angabe von Gründen die Löschung bereitgestellter Daten verlangen können. Für eine transparente und effektive Umsetzung wird hierfür im GestaltMatcher-Projekt eine Blockchain-Technologie eingesetzt. Dabei handelt es sich um eine verteilte Datenbank ohne zentrale Verwaltung, die durch Verkettung ihrer Datensätze vor Manipulation gesichert ist. Beim GestaltMatcher ist sie öffentlich einsehbar und es können sogenannte *Smart Contracts *(digitale Verträge in Form von Programmen innerhalb der Blockchain) definiert werden, die zum Beispiel sicherstellen, dass Daten nicht den europäischen Raum verlassen.

## Fazit

Mit weiteren Verbesserungen in der Sequenziertechnik wird die wesentliche Verzögerung in der Diagnosestellung von Seltenen Erkrankungen nicht am Labor scheitern, sondern an der Indikationsstellung für den genetischen Test. Software-Anwendungen mit künstlicher Intelligenz können zu einer Verbesserung in der Diagnostik von Seltenen Erkrankungen führen, indem sie das Wissen über pathognomonische Muster in medizinischen Bilddaten wie Porträtbildern einem breiten Expertenspektrum, also auch Kinderärzt:innen und Neurolog:innen, zugänglich machen. Das Vertrauen in den Einsatz von KI wird steigen, wenn Patient:innen auf einfache Weise entscheiden können, wer zur welchem Zweck Zugang zu ihren Daten erhält, und wenn sie nachvollziehen können, warum eine Ärztin oder ein Arzt zu welcher Schlussfolgerung kommt. Ärzt:innen werden durch KI nicht ersetzt, sondern es wird ihnen ermöglicht, die Qualität ihrer Arbeit zu steigern.

## Footnotes

1 https://hpo.jax.org/app/browse/term/HP:0001999.
